# Person-centered care and access to drugs in the digital environment: to cure or to sell

**DOI:** 10.3325/cmj.2019.60.174

**Published:** 2019-04

**Authors:** Vanja Kopilaš

**Affiliations:** Croatian Studies, University of Zagreb, Zagreb, Croatia *vkopilas@hrstud.hr*

## Modern era

Technology plays an important role in our daily lives, making us more efficient but also increasingly technology dependent. Technology, and in particular digital technology, is omnipresent, and the list of ways in which it has affected our lives is long. This is especially true when considering how much technology has improved medicine. Robotic surgeries and artificial organs are no longer the imagery of science-fiction movies, but are implemented in medicine on a daily basis. However, the advantages of technology have made us overlook or push aside its many drawbacks. Therefore, it is critical to examine both the beneficial and the negative effects of technology in medicine, and to understand the less visible bonds between these two fields.

One of the aims of Knowledge Landscapes is to narrow the gap between available resources and patients by empowering them with the right tools to interpret information correctly (1). When faced with the abundance of information available online, it is up to the individual to decide how to use that information. Patients now approach physicians with their own knowledge about their illnesses and have caused pharmaceutical companies to rethink their strategy and involve patients in the development of medications and processes, such as adherence, access, administration methods, and marketing (2). In the digital world, patients are exposed to information about their condition, which is curated by professionals, but also filtered according to the purpose. This is where it becomes tricky. Although online resources can be very useful, problems may arise from the fact that these resources present selective, simplified, and biased information. Furthermore, patients increasingly take that information at face value, and use it to make potentially harmful health-related decisions. Needless to say, these ill-informed decisions can lead to dangerous and chaotic outcomes.

## Dangers of technology: providing drugs through internet-based platforms

Due to high demand for prescribed medications, their abuse has become a growing issue (3). High demand is accompanied by services trying to meet it. Technology, or more precisely, interned-based platforms, perfectly fit into this role. They serve as mediators between patients (or, in this case, customers) and their medication. Historically, physicians were responsible for choosing which medications to prescribe, but even this has shifted to benefit the market. Physicians have become affected by the business model of drug sales, which has influenced and sometimes overpassed the ethical and safety norms. Even though their role in prescribing medications has remained unchanged, physicians are still impacted by this technological shift. For example, recent surveys suggest that over 70% of patients in the United States would prefer virtual doctor visits (4). Thus, physicians are in a way expected to treat patients both in person and digitally. Furthermore, physicians frequently have some level of competing interests that are difficult to discern, sometimes hidden in the complex financial interdependencies of the industry, health care system, and individual professionals. In the more extreme cases, physicians are employees of companies that are in charge of the selling platforms and it is in physicians’ interest to prescribe more medications. More prescribed medications means more bonuses, creating a setting where financial interests could bias the professional decisions. It is worth noting that this is not only about physicians’ morality, but that it affects a more significant area: it has clear consequences on patients’ health.

Insisting on high standards of medical professionals is not a matter of pure formality, but rather a matter of patients' safety and well-being. According to recent statistics in the United States, drug overdoses have caused more fatalities than car accidents and gun violence (5). Taking all this into account, the way in which patients are prescribed medications is also an important factor to consider. Certain digital applications allow patients to complete an online health-related questionnaire, based on which physicians prescribe medications to them (6). By prescribing medications based solely on online questionnaires, professionals do not contribute to the quality of patient-centered care. Subsequently, relying on the digital environment does not empower patients by providing them the right tools and helpful resources.

## Digital media misrepresentation

In addition to the development and accessibility of medications, their marketing and media representation should also be addressed. Digital media present on the internet are considerably less controlled and regulated than their non-internet counterparts, such as television and magazines. Every so often, there are texts glorifying and maximizing miraculous effects of some substances. By promoting the unproven or untested substances, these platforms minimize and devalue numerous years of research invested in the development of real medications. Professionals can easily discredit these falsely represented declarations, but the vast majority of people cannot. People can become manipulated by these messages, with no real reason to doubt their accuracy. Research has shown that media misrepresentation of health risks was associated with higher perceptions of the severity and prevalence of illnesses (7,8). Therefore, it is crucial to be vocal and accurate during the information exchange process. This is why Knowledge Landscapes’ goal is to make it understandable how knowledge is represented and interpreted to benefit the users (9). Implementing stricter guidelines, which would ensure that advertisements are clearly labeled as such, would help consumers navigate through online materials without being misled to believe advertisements are scientifically based. A review of antidepressant marketing materials shows how consumers are exposed to selectively chosen information (10). There is a huge disparity between advertisements and scientific literature. Advertisements emphasize the positive effects of antidepressants, while their side effects are minimized or even not mentioned (10). Simply put, antidepressants are presented as magic pills that alleviate all symptoms. These misrepresentations impact a range of audiences from physicians to patients. Physicians are not immune to the flashy and tempting advertisements and may, on some level, be influenced to buy into the marketing tactics. Perhaps, the promise of a new medication that can improve health conditions intrigues physicians to prescribe them to their patients. Since marketing affects physicians, it is no surprise that it also affects patients, who rely on expert opinion. People who are unfamiliar with the aspects of depressive symptoms, mechanisms that maintain them, and effects of treatment may be convinced that magic pills exist. This brings to question the morality of presenting potential “consumers” just one side of the story, while they are led to believe they are well informed.

## Conclusion

As technology continues to thrive, its effects on modern-day medicine range from beneficial to potentially harmful. The development of available medications, and the ways in which they are accessed and marketed, clearly shows the negative and morally questionable impact of the digital environment on person-centered care. Without a proper channel that provides consumers with accurate information, misinformation regarding medications can have detrimental effects. More effort should be invested in the creation of proper channels that regulate health-related materials on internet platforms, resulting in a better health system with minimized risks and maximized benefits.
